# Effects on Liver Lipid Metabolism of the Naturally Occurring Dietary Flavone Luteolin-7-glucoside

**DOI:** 10.1155/2015/647832

**Published:** 2015-05-31

**Authors:** Carla Sá, Ana Rita Oliveira, Cátia Machado, Marisa Azevedo, Cristina Pereira-Wilson

**Affiliations:** ^1^Department of Biology, School of Sciences, University of Minho, 4710-057 Braga, Portugal; ^2^Center for the Research and Technology of Agro-Environmental and Biological Sciences (CITAB), Department of Biology, School of Sciences, University of Minho (UM), 4710-057 Braga, Portugal

## Abstract

Disruptions in whole-body lipid metabolism can lead to the onset of several pathologies such as nonalcoholic fatty liver disease (NAFLD) and cardiovascular diseases (CVDs). The present study aimed at elucidating the molecular mechanisms behind the lipid-lowering effects of the flavone luteolin-7-glucoside (L7G) which we previously showed to improve plasma lipid profile in rats. L7G is abundant in plant foods of Mediterranean diet such as aromatic plants used as herbs. Results show that dietary supplementation with L7G for one week induced the expression of peroxisome proliferator-activated receptor-alpha (PPAR-*α*) and of its target gene carnitine palmitoyl transferase 1 (CPT-1) in rat liver. L7G showed a tendency to decrease the hepatic expression of sterol regulatory element-binding protein-1 (SREBP-1), without affecting fatty acid synthase (FAS) protein levels. Although SREBP-2 and LDLr mRNA levels did not change, the expression of HMG CoA reductase (HMGCR) was significantly repressed by L7G. L7G also inhibited this enzyme's *in vitro* activity in a dose dependent manner, but only at high and not physiologically relevant concentrations. These results add new evidence that the flavone luteolin-7-glucoside may help in preventing metabolic diseases and clarify the mechanisms underlying the beneficial health effects of diets rich in fruits and vegetables.

## 1. Introduction

Nonalcoholic fatty liver disease (NAFLD) is one of the major causes of liver disease and is linked to the obesity and metabolic disease epidemics. NAFLD is characterized by excessive lipid accumulation in the liver accompanied by metabolic dysfunction and hepatic cell degeneration. NAFLD is considered to be the liver manifestation of insulin resistance. Hyperlipidaemia (increased blood triglycerides and/or cholesterol) is frequently associated with metabolic diseases and is also associated with fatty liver disease [1]. Lu et al. (2013) suggest that NAFLD is a strong independent predictor of cardiovascular diseases (CVDs) [2].

**Figure 1:**
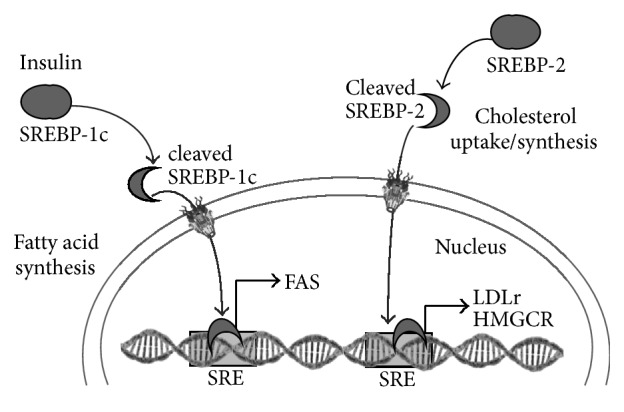
Schematic representation of the sterol receptor element-binding protein- (SREBP-) 1c and 2 transcription factors and respective target genes involved in fatty acid or cholesterol synthesis and uptake, respectively. SREBP-1c is activated in response to insulin and binds to sterol response elements (SRE) inducing target genes such as fatty acid synthase (FAS), while SREBP-2 activation induces 3-hydroxy-3-methyl-glutaryl-CoA reductase (HMGCR) and the low density lipoprotein receptor (LDLr) genes.

While in adipose tissue insulin resistance leaves lipolysis uninhibited, in the liver insulin resistance decreases its ability to downregulate gluconeogenesis without affecting lipogenic responses mediated by sterol regulatory element-binding protein-1c (SREBP-1c), including induction of fatty acid synthase (FAS) (Figure 1). The result is increased lipids in circulation and in the liver where lipogenesis continues to take place [3, 4]. Also increased, hepatic SREBP-2 activation leads to expression of its target genes 3-hydroxy-3-methyl-glutaryl-CoA reductase (HMGCR) and the low density lipoprotein receptor (LDLr) (Figure 1) [5]. These genes regulate cholesterol metabolism and play an important role in the lipoprotein profile in the blood. Inhibition of HMGCR activity results in decreased LDL plasma levels and is the mechanism of action of statins, pharmaceutical drugs used in the prevention of CVD [6].

**Figure 2:**
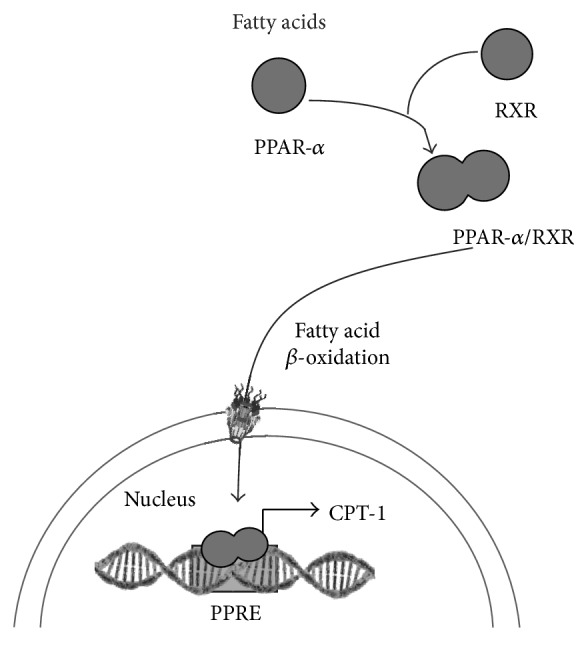
Schematic representation of heterodimers of peroxisome proliferator-activated receptor-*α* and retinoid X receptor (PPAR-*α*/RXR) binding to specific PPAR responsive elements (PPRE), which promote *β*-oxidation through increasing the expression of carnitine palmitoyl transferase 1 (CPT-1).

Liver lipid metabolism is in addition regulated by peroxisome proliferator-activated receptor-alpha (PPAR-*α*) that activates fatty acid *β*-oxidation, decreasing liver lipid deposits through carnitine palmitoyl transferase 1 (CPT-1) activation (Figure 2) [7, 8]. The involvement of PPAR-*α* activity in NAFLD has been demonstrated in experiments with knockout animals that develop hepatic steatosis [9]. Fibrates are pharmacological agonists of PPAR-*α* used in the control of dyslipidemias.

Nutrition is a decisive factor in general human health and disease. A link has been demonstrated between increased caloric intake and a sedentary life style with metabolic syndrome, insulin resistance, dyslipidemia, and NAFLD [10, 11]. Low intake of antioxidants and micronutrients has also been shown to contribute to the development of these diseases [12, 13].

As oxidative damage is a major contributor to injury in the fatty liver, antioxidant supplementation has often been suggested to be beneficial. Vitamins and plant food flavonoids may, therefore, offer protection against NAFLD and be valuable in the prevention of the disease.

Flavonoids are a large group of natural compounds with antioxidant properties present in vegetables, fruits, and aromatic plants [14]. High dietary intake of fruits, vegetables, and whole grains is strongly connected with a lower risk of developing cancer, cardiovascular disease, and other chronic diseases with high prevalence [15].

Flavonoids can be divided according to the degree of oxidation of the oxygen heterocycle and the substitution patterns in 6 main subclasses: anthocyanins (cyanidin, malvidin, pelargonidin, and peonidin), flavanones (hesperidin, naringenin), flavan-3-ols (catechins, epicatechin, epigallocatechin, and gallocatechin), flavones (luteolin, apigenin, and chrysin), flavonols (kaempferol, myricetin, quercetin, and rutin), and isoflavones (daidzein, genistein, and glycitein) [16–18].

A recent study on the effects of flavonoid intake on cardiovascular disease mortality found that flavone consumption was associated with the greatest reduction in cardiovascular diseases in women (other flavonoids classes used in the study were anthocyanidins, flavan-3-ols, flavanones, and flavonols) and that flavone consumption was associated with the lowest risk of fatal ischemic heart disease, in both men and women [19].

**Figure 3:**
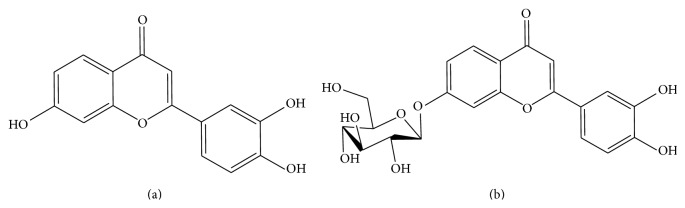
Chemical structures of the flavones luteolin (a) and luteolin-7-glucoside (b).

Luteolin (L) (Figure 3(a)) and its glycosylated form luteolin-7-glucoside (L7G) (Figure 3(b)) belong to the flavone subclass of flavonoids and are among the most common flavonoids present in aromatic plants and other plant foods consumed in the Mediterranean diet (Table 1) [20–22]. Generally, flavones are present in their glycosylated forms. Glycosylation increases their solubility in the aqueous cellular environment allowing storage in the cell's vacuoles [14, 23]. Although glycosylated forms are the most common in nature it has been established that luteolin is absorbed in the aglycone form only [24] which raises questions related to the efficacy of L7G as a dietary constituent. Aside from its well-known antioxidant and anticarcinogenic properties [25–29], the lipid-lowering potential of luteolin has been recently demonstrated in palmitate-stimulated HepG2 cells [30]. The authors found a reduction of SREBP-1c and FAS expression and an increase of CPT-1 expression, as a consequence of the luteolin-mediated AMPK activation and ROS inhibition [30]. Our lab has previously shown that L7G given in the diet for 1 week improved plasma lipid profile in rats [31]. Here we demonstrate that these effects are accompanied by an increase in liver expression of fatty acid *β*-oxidation related genes (PPAR-*α* and CPT-1) as well as a decrease in the rate limiting enzyme of cholesterol synthesis (HMGCR). Liver lipogenesis (SREBP-1 and FAS levels) was not modified by L7G treatment. These effects are consistent with the decrease in LDL and total cholesterol observed in the plasma of animals fed with L7G and indicate that this compound may, in addition to improving plasma lipid profile, be beneficial in the prevention of NAFLD and other metabolic diseases.

## 2. Materials and Methods

### 2.1. Reagents and Antibodies

Luteolin-7-O-glucoside was purchased from Extrasynthese (Genay, France). Antibodies against FAS and secondary antibody HRP (containing IgG horseradish peroxidase) goat anti-rabbit were acquired from Cell Signaling (Danvers, MA, USA). Antibodies against caspase-3, total- and phospho-JNK, and secondary antibody HRP goat anti-mouse were bought from Santa Cruz Biotechnology, Inc. (Santa Cruz, CA, USA). The iScript cDNA Synthesis Kit and SsoFast EvaGreen supermix utilized in quantitative real-time PCR as well as the DC protein assay kit used in protein concentration determination were acquired from Bio-Rad (Bio-Rad Laboratories, Inc., Hercules, CA, USA). TRI Reagent, HMG-CoA reductase assay kit, anti-*β*-actin antibody, and all other reagents were of analytical grade and acquired from Sigma-Aldrich (St. Louis, MO, USA).

### 2.2. Animals

Six-week-old male Wistar rats were purchased from Charles River Laboratories (Barcelona, Spain) and acclimated for at least one week before the start of the experiment, in authorized animal facilities of the Life and Health Sciences Research Institute from University of Minho. During experiments animals had* ad libitum* access to food and tap water and were maintained under controlled conditions of temperature (20 ± 2°C) and humidity (55 ± 10%), with a 12 h light/12 h dark cycle. Animals were kept in accordance with the NIH guidelines for the experimental use and care of laboratory animals and handled by authorized investigators by the Direcção Geral de Veterinária, Portugal. The experiment was approved by university's ethics committee that follows NIH guidelines (NIH Publication number 80-23, revised 1978) for use and care of laboratory animals.

### 2.3. Experimental Design

Male rats were divided into two distinct groups and fed a L7G supplemented diet and control diet. In L7G supplemented diet group, rats receive 2 mg of L7G per kg of animal body weight mixed in a small piece of food, once a day, for 7 consecutive days; control group received the control diet. The amount of L7G administered was based on human luteolin intake estimations by Hertog and collaborators in 1993 [39]. Supplementation with L7G did not alter animal's body weight or food and beverage consumption when compared to control group. At the end of experiment, animals were sacrificed by decapitation. Liver samples were collected, frozen in liquid nitrogen, and kept at −80°C until further analysis.

### 2.4. RNA Extraction and Quantitative Real-Time PCR

Total RNA was isolated from frozen liver samples using TRI Reagent (Sigma-Aldrich), according to manufacturer's instructions. RNA quantification was performed using NanoDrop ND-1000 spectrometer, and its purity was confirmed by A260/A280 ratio. Conversion of RNA into cDNA was performed using iScript cDNA Synthesis Kit (Bio-Rad) following the producer's suggested protocol. Prior to gene expression analysis, the integrity of cDNA was confirmed by agarose gel electrophoresis.

Quantitative gene expression analysis was performed using SYBR Green technology (SsoFast EvaGreen supermix) and CFX96 real-time system from Bio-Rad. Samples were amplified using the following conditions: an initial denaturation step of 3 min at 95°C followed by 40 cycles of 10 s at 95°C and 30 s at 60°C. The primers used were manufactured by STAB VIDA, Lda (Caparica, Portugal) and sequences are presented in Table 2.

Each assay included a relative standard curve constructed from serial dilutions of cDNA from control samples. Target genes' transcript levels were all normalized to *β*-actin mRNA levels and expression values in the control group were set to 1.

### 2.5. Liver Homogenates

A small amount of liver was homogenized in cold lysis buffer (0.5% NP-40 in 50 mM Na_2_HPO_4_, pH 7.4, 150 mM NaCl_2_, and 2 mM EDTA) supplemented before use with protease and phosphatase inhibitors (1 mM phenylmethylsulfonyl fluoride, 10 *μ*g/mL aprotinin and 20 mM NaF, and 20 mM Na_3_VO_4_). The homogenate was centrifuged at 10000 ×g at 4°C for 10 min and the supernatant collected. Protein concentration was determined using Bio-Rad DC protein assay kit (Bio-Rad) according to manufacturer's instructions using BSA as standard. Samples were kept at −80°C for further analysis.

### 2.6. Western Blot Analysis

Proteins in each sample were separated by SDS-PAGE and transferred onto Hybond-P polyvinylidene difluoride membranes (GE Healthcare, Buckinghamshire, UK). Membranes were blocked in 5% (w/v) nonfat dry milk in TPBS (0.05% (v/v) Tween 20 in PBS, pH 7.4) and then washed in TPBS and incubated overnight with primary antibody. After washing with TPBS, membranes were incubated with secondary antibody and immunoreactive bands were detected using the Immobilon solutions (Millipore, Billerica, MA, USA) under an imaging densitometer (ChemiDoc XRS from Bio-Rad). Band area intensity was quantified using the Quantity One software (Bio-Rad). *β*-actin was used as loading control.

### 2.7. HMGCR Activity* In Vitro* Assay

The* in vitro* effect of L7G at 10, 30, and 100 *μ*M on HMGCR enzyme activity was determined using a HMG-CoA reductase assay kit from Sigma-Aldrich, as recommended by manufacturers. L7G was dissolved in DMSO (final concentration in the assays of 0.5% (v/v)); DMSO was used as control and pravastatin, a commercial statin, at 0.5 *μ*M was used as a positive control.

### 2.8. Statistical Analysis

Data are presented as means ± SEM. For statistical analysis GraphPAD Prism 5.0 software (San Diego, CA, USA) was used. Student's *t*-test was utilized to compare differences between control and L7G treatment. *P* values ≤0.05 were considered statistically significant.

## 3. Results and Discussion

Diets rich in fruits and vegetables have long been associated with health benefits attributed at least in part to the high antioxidant levels in these diets. Flavonoids are important dietary antioxidants but may exert their health improving benefits by mechanisms other than by conferring increased antioxidant defense.

In previous studies, we have described the beneficial effects of a luteolin-7-glucoside (L7G) rich herbal tea prepared from the aromatic plant* Salvia officinalis* (SO) on human plasma lipoproteins [40]. The decrease in total and LDL cholesterol observed in humans was significant and similar to the effects of L7G on plasma lipoproteins of L7G fed rats [31]. Additionally, SO showed metformin-like effects in rat hepatocyte primary cultures [41].

Overall these previous data suggested that L7G, the major flavone constituent in SO but also abundant in other herbs and plant foods (Table 1), might help to regulate liver lipid metabolism and help in the dietary control of NAFLD and other metabolic diseases.

In the present study we analyzed the effects of the flavone luteolin in its naturally more abundant glycosylated form, L7G, on the modulation of regulators of liver lipid metabolism* in vivo*.

**Figure 4:**
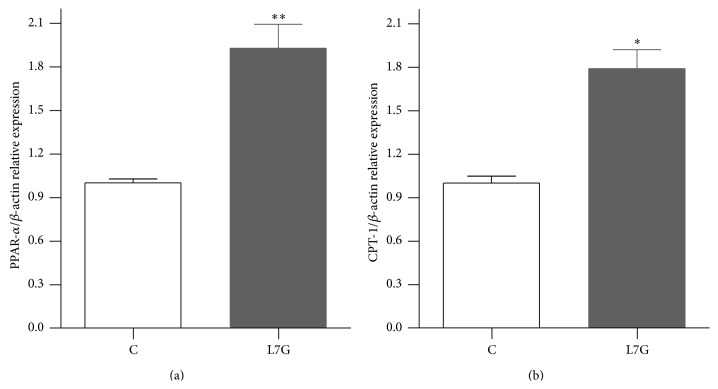
Effects of L7G supplemented diet on rat liver PPAR-*α* (a) and CPT-1 (b) gene expression. Results are presented as relative expression to the control. Values are means ± SEM and *n* = 5. ^*∗*^ *P* ≤ 0.05 and ^*∗∗*^ *P* ≤ 0.01 when compared with the control group.

**Figure 5:**
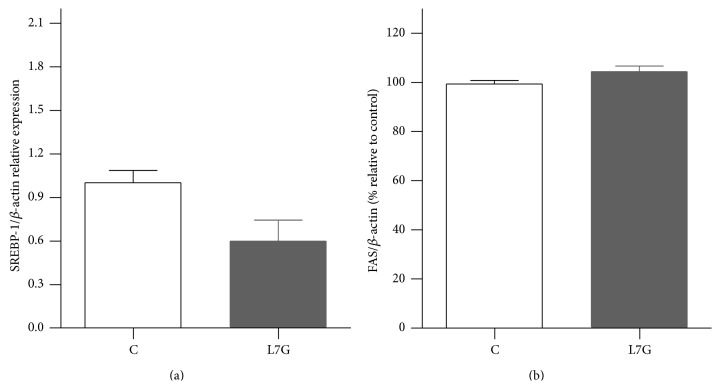
The effects of L7G supplemented diet on rat liver SREBP-1 (a) gene expression and western blot analysis of FAS protein levels (b). Results are presented as relative expression to the control. Values are means ± SEM and *n* = 5.

Rats were fed a diet supplemented with L7G (2 mg/kg body weight) for 1 week. The introduction of this flavone in the diet was well tolerated and no changes in animal or liver weight were found. The effects of L7G on hepatic lipid metabolism were analyzed by quantitative real-time PCR (qPCR) of genes involved in fatty acid metabolism (PPAR-*α* and CPT-1), lipid synthesis (SREBP-1), and cholesterol metabolism (SREBP-2, HMGCR, and LDLr). FAS protein levels were analyzed by western blot. Livers of L7G treated animals showed significantly increased (1.9-fold) mRNA levels of PPAR-*α* and of its target gene, CPT-1 (1.8-fold) (Figures 4(a) and 4(b)). Only a slight decrease in SREBP-1 expression was detected and no effect seemed to be present on FAS protein levels (Figures 5(a) and 5(b)).

Taken together our data indicates an increased *β*-oxidation capacity in the liver in response to dietary L7G without significant effects on lipogenesis. L7G's effect on activation of PPAR-*α* suggests a fibrate-like mechanism of action. Fibrates are pharmaceutical drugs used to treat dyslipidemia and are known PPAR-*α* ligands (activators). Their use has also been suggested in the treatment of NAFLD [42]. Fibrates seem to improve some but not all features of NAFLD in patients with the disease. Our data suggests that L7G, as a dietary constituent, tends to decrease liver lipid deposition and might be useful in the prevention of NAFLD, although further studies are required to verify this possibility.

**Figure 6:**
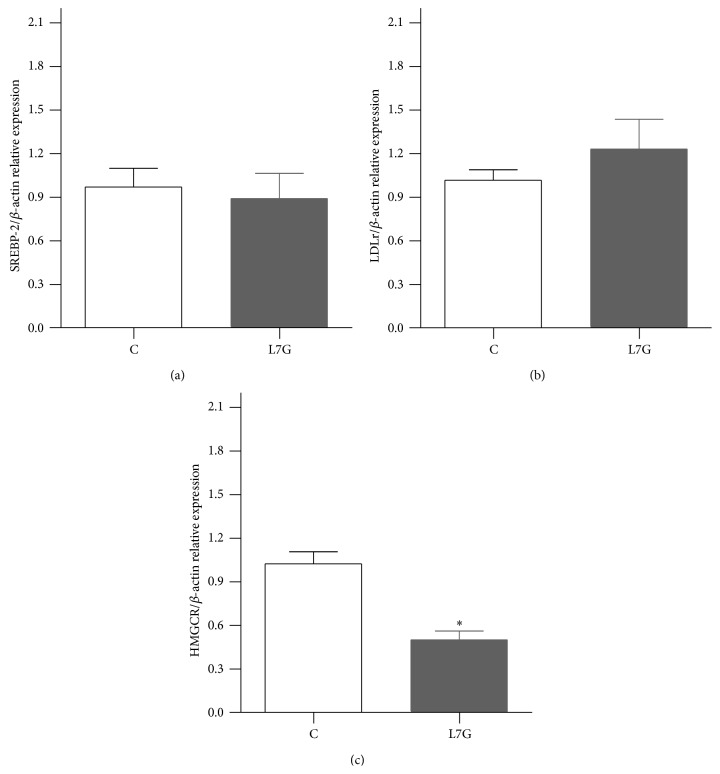
The effects of L7G supplemented diet on rat liver SREBP-2 (a), LDLr (b), and HMGCR (c) gene expression. Results are presented as relative expression to the control. Values are means ± SEM and *n* = 5. ^*∗*^ *P* ≤ 0.05 when compared with the control group.

**Figure 7:**
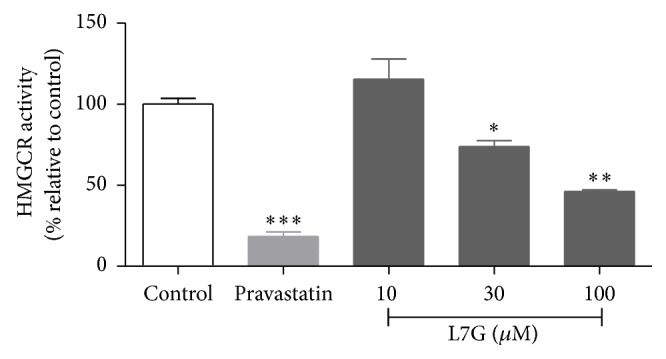
*In vitro* effect of L7G on HMGCR enzyme activity. Pravastatin (0,5 *μ*M), a commercial statin inhibitor of the enzyme, was used as positive control. Values are means ± SEM and *n* = 3. ^*∗*^ *P* ≤ 0.05, ^*∗∗*^ *P* ≤ 0.01, and ^*∗∗∗*^ *P* ≤ 0.001 when compared to the control.

The hepatic expression of SREBP-2 and of its target gene LDLr was not significantly changed by L7G (Figures 6(a) and 6(b)). The expression of HMGCR was, however, significantly decreased in the L7G treated group (Figure 6(c)). HMGCR, another target of SREBP-2, is the rate limiting enzyme of cholesterol synthesis and its decreased expression is in agreement with the previously observed decrease in total plasma cholesterol and LDL lipoprotein levels [31]. The observed decreased expression of SREBP-2 dependent HMGCR suggests decreased cholesterol synthesis by an alternative mechanism to that of statins that inhibit the enzyme's activity. The direct effect of L7G on HMGCR activity was tested* in vitro* (Figure 7) and the results show that L7G is not a good inhibitor of this enzyme at physiologically relevant concentrations, when compared to pravastatin.

Interestingly, both statins and the combination of statins and fibrates have been shown to be useful in the management of NAFLD [42–44]. This combination of effects induced by L7G alone suggests that L7G rich diets may be useful in the control of NAFLD and other metabolic diseases. With regard to the effects on lipoproteins of animals fed a diet supplemented with L7G where a significant decrease in LDL (39.5%) and total cholesterol (29.2%) was observed and in humans where the herbal tea of* Salvia officinalis* also significantly improved plasma lipid profile, reducing LDL (by 12.4%) and increasing HDL (by 50.6%) [31, 40], it seems that L7G is a major active compound capable of regulating liver lipid metabolism and producing beneficial effects against NAFLD and CVD.

Importantly, the liver of animals fed the L7G supplemented diet did not show increases in JNK or cleaved caspase-3 levels (data not shown). Hepatic cell degeneration due to increased apoptosis is a feature of NAFLD mediated by oxidative stress, inflammatory cytokines, and activation of the JNK signaling pathway [45–47]. JNK activation is also involved in insulin resistance [48]. Our results show that L7G does not induce activation of the JNK pathway or increase liver apoptosis. These pathways were, however, not originally increased in our experimental model where healthy animals were used.

## 4. Conclusions

In addition to its well-known antioxidant properties, the dietary flavone L7G promotes liver lipolysis through induction of PPAR-*α* and CPT-1 expression. L7G also decreases HMGCR expression thereby decreasing cholesterol synthesis and contributing to lower total and LDL cholesterol levels. This combination of effects induced by L7G suggests that L7G rich diets may be useful in the control of both NAFLD and CVD.

## Figures and Tables

**Table 1:** Aromatic and other plant foods rich in luteolin and luteolin-7-glucoside^*∗*^.

Flavone form	Aromatic plants	Other plants and vegetables
Luteolin	Oregano, *Origanum vulgare* Parsley, *Petroselinum crispum* Thyme, *Thymus vulgaris* Perilla, *Perilla frutescens *	Olives, *Olea europaea* Artichokes, *Cynara scolymus* Carrots, *Daucus carota* Pomegranate, *Punica granatum* Cacao, *Theobroma cacao* Rooibos (herbal tea), *Aspalathus linearis* Turnip, *Brassica napus *

Luteolin-7-glucoside	Sage, *Salvia officinalis* Parsley, *Petroselinum crispum* Thyme, *Thymus vulgaris *	Olives, *Olea europaea* Artichokes, *Cynara scolymus* Celery, *Apium graveolens* Chayote, *Sechium edule* Cacao, *Theobroma cacao* Capers, *Capparis spinosa *

Luteolin and unidentified glycosylated forms	Anise, *Pimpinella anisum* Chervil, *Anthriscus cerefolium *	Broccoli, cauliflower, and cabbage, *Brassica oleracea* Spinach, *Spinacia oleracea* Peppers, *Capsicum annuum* Peppermint, *Mentha piperita* Tamarind, *Tamarindus indica* Chamomile (herbal tea) Red grapes, *Vitis vinifera* Lemon, *Citrus limon* Radicchio, *Cichorium intybus* Pumpkin, *Cucurbita *spp.

^*∗*^Information in [20–22].

**Table 2:** Primers sequences used in quantitative real-time PCR analysis.

Gene	Sequences	Product size (bp)	Efficiency	References
SREBP-1	Forward: AGCGCTACCGTTCCTCTAT	95	2.10	[32]
	Reverse: GCGCAAGACAGCAGATTTAT			

SREBP-2	Forward: ATTCCCTTGTTTTGACCACGC	248	2.10	[33]
	Reverse: TGTCCGCCTCTCTCCTTCTTTG			

PPAR-*α*	Forward: GATTCGGAAACTGCAGACCTC	444	2.01	[34]
	Reverse: TAGGAACTCTCGGGTGATGA			

CPT-1	Forward: CAGGATTTTGCTGTCAACCTC	162	2.10	[35]
	Reverse: GAGCATCTCCATGGCGTAG			

LDLr	Forward: GCATCAGCTTGGACAAGGTGT	114	2.05	[36]
	Reverse: GGGAACAGCCACCATTGTTG			

HMGCR	Forward: AGTGATTGTGTCAGTATTATTAGTGGAAG	91	2.00	[37]
	Reverse: GGTACTGGCTGAAAAGTCACAA			

*β*-actin	Forward: AGAGGGAAATCGTGCGTGAC	138	2.04	[38]
	Reverse: CAATAGTGATGACCTGGCCGT			
